# The homeodomain transcription factor Phox2 in the stellate ganglion of the squid *Loligo pealei*

**DOI:** 10.1242/bio.012476

**Published:** 2015-06-26

**Authors:** J. Peter H. Burbach, Anita J. C. G. M. Hellemons, Philip Grant, Harish C. Pant

**Affiliations:** 1 Brain Center Rudolf Magnus, Department of Translational Neuroscience, University Medical Center Utrecht, Utrecht University, Utrecht 3584CG, The Netherlands; 2 Marine Biological Laboratory, Woods Hole, MA 02543, USA; 3 Laboratory of Neurochemistry, National Institute of Neurological Disorders and Stroke, Bethesda, MD 20892, USA

**Keywords:** Homeodomain transcription factors, Squid, Phox2, FMRFamide, Stellate ganglion, Squid giant axon, Brain development

## Abstract

Homeodomain transcription factors regulate development of embryos and cellular physiology in adult systems. Paired-type homeodomain genes constitute a subclass that has been particularly implicated in establishment of neuronal identity in the mammalian nervous system. We isolated fragments of eight homeodomain genes of this subclass expressed in the stellate ganglion of the North Atlantic long finned squid *Loligo pealei* (*lp*) [Note: *Loligo pealei* has been officially renamed *Doryteuthis pealei*. For reasons of uniformity and clarity *Loligo pealei* (*lp*) is used here]. Of the most abundant ones, we cloned a full length cDNA which encoded the squid ortholog of the paired-type homeodomain proteins Phox2a/b. The homology of lpPhox2 to invertebrate and mammalian Phox2 was limited to the homeodomain. In contrast to mouse Phox2b, lpPhox2 was unable to transactivate the *dopamine beta-hydroxylase* (*DBH*) promoter in a heterologous mammalian transfection system. *In vivo*, *lpPhox2* was expressed in the developing stellate ganglion of stage 27 squid embryos and continued to be expressed in the adult stellate neurons where expression was confined to the giant fiber lobe containing the neurons that form the giant axons. The expression of *lpPhox* was similarly timed and distributed as the *Fmrf* gene. Furthermore, the *Fmrf* upstream region contained putative Phox2a/b binding sites. These results suggest a role of *lpPhox2* in the developmental specification of neuronal identity and regulation of neurons of the squid giant axon.

## INTRODUCTION

The development of the nervous system involves multiple stages of cellular commitment that lead via patterning, proliferation, and fate determination to defined neuronal identities. These processes are driven by complex regulatory networks of transcription factors and signalling molecules (Andersson et al., 2006; Dasen et al., 2005; Jessell, 2000; Smidt and Burbach, 2007). Neuronal identity is often marked by a neurotransmitter phenotype, as well as its specific connectivity to other neurons. These two properties allow neurons to function in neural networks and to contribute specific circuitry.

Several families of transcription factors have been demonstrated to play a key role in the development of the mammalian nervous system. Early stages of commitment to neural cell fates and proliferation involve basic helix-loop-helix factors, while homeodomain proteins provide patterning and neuronal fate specification (Goridis and Brunet, 1999; Guillemot, 2007; Smidt and Burbach, 2007). For example, homeodomain proteins encoded by the Hox clusters in spinal cord, that are expressed in a segmental fashion, are involved in pattern formation (Dasen et al., 2005; Pearson et al., 2005; Trainor and Krumlauf, 2001). Non-Hox homeodomain genes participate in further stages of differentiation in all parts of the nervous system, mostly confined to specific cell groups that share neuronal identity (Asbreuk et al., 2002; Molyneaux et al., 2007). Such factors often retain expression in the mature nervous system and adopt regulatory functions (Davis et al., 2010; Zhu et al., 2005a). In the mammalian nervous system processes driven by homeodomain factors result in the development of highly complex neural structures. In the more simple nervous systems of lower vertebrates and invertebrates similar cascades of transcription factors also operate during nervous system development (Hammock et al., 2010; Sen et al., 2013). This suggests that functions of homeodomain factors are generic and may play a role in all nervous systems of the animal kingdom (Marder, 2002).

In the mammalian nervous system neuronal diversity is enormous so as to produce a nervous system that can meet the complex demands of the organism. In order to evaluate homeodomain gene function in the establishment of neuronal identity and connectivity, we turned to neurons that are more homogenous in anatomy and function, namely, those of the squid giant axon (Marder, 2002). The squid giant axon is a classical preparation in which fundamental features of neurons, such as cellular anatomy, axonal transport, control of membrane potentials and ion currents, and neurotransmission were first explored (Hodgkin and Huxley, 1952; Llinas, 1988; Webb and Young, 1940; Young, 1939). The squid giant axon is a thousand-fold larger in diameter than mammalian axons because it is formed by the fusion of axons from multiple neurons located in the giant fiber lobe of the stellate ganglion (Young, 1939). The ganglion contains two types of neurons. One set of small neurons in the giant fiber lobe provides axons which fuse into the giant axons that innervate the mantle muscles. The axons of these neurons, when excited by axo-axonic contacts, drive jet propulsion as part of the escape response of the squid (Gilly et al., 1990). In addition, they control water flow over the gills, serving a respiratory function. The second set consists of large neurons that control fin movement and fine control of swimming. The anatomy of the stellate ganglion and its afferent connectivity with the central nervous system of the squid have been extensively detailed in the species *Loligo pealei* since the 1930s (Young, 1939, 1973).

The involvement of Hox clusters in the outline of the embryonic body plan of the squid has been demonstrated (Arnold, 1990; Lee et al., 2003; Martin, 1965). However, the molecular mechanisms of neuronal fate determination are unknown. To explore such mechanisms in the squid, we here aimed to identify and characterize homeodomain transcription factors expressed in the squid stellate ganglion. To this end a RT-PCR cloning strategy was adopted to identify paired-like homeodomain transcription factors in the stellate ganglion (Asbreuk et al., 2002), and the most abundant one was cloned and characterized with respect to expression and transcriptional activity. The results show that this factor is the squid homologue of Phox2 and that it may have a potential function in developmental specification of neuronal identity and regulation in the squid stellate ganglion.

## RESULTS

### Identification of homeodomain transcripts

To screen for homeobox genes expressed in the stellate ganglion we employed a PCR cloning strategy used previously to identify paired-type homeobox genes expressed in rodent brain (Asbreuk et al., 2002). Sequencing of clones identified eight PCR fragments of 120 nucleotides with similarity to mammalian homeodomain sequences. Similarities indicated that these were squid orthologs of the homeobox genes Msx1/2/3, Pitx2/3, Phox2a/b, ARX/Gsc, Nkx1, zinc finger homeodomain protein-4, and POU6-2/RPF-1 (supplementary material). Screening of a cDNA phage library constructed from adult stellate ganglia by filter hybridization, using labelled PCR fragments as probes, resulted in the isolation of clones for RPF-1 and Phox2, but none of the other homeodomains.

### Structural properties of lpPhox2

**Fig. 1.**
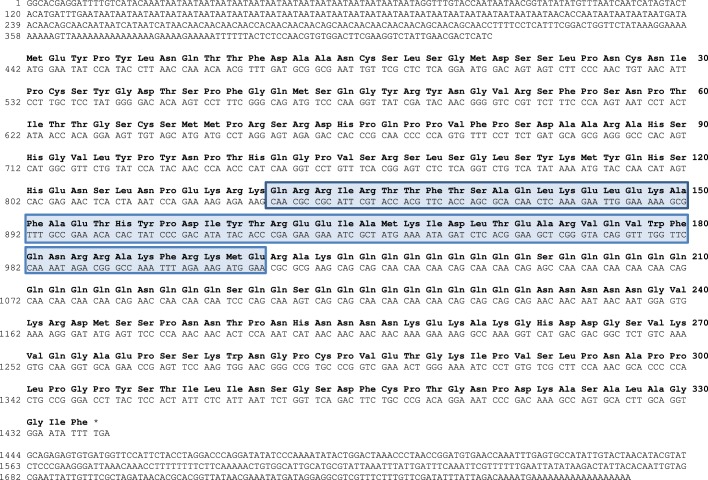
**Primary structure of lpPhox2.** Amino acid sequence of the homeodomain proteins lpPhox2 from a 1790 nt cDNA (accession number KT159250). The open reading frame of the cloned transcript encodes a protein of 333 amino acids with the 60 amino acid homeodomain between residues 132 and 191 (boxed).

A clone with a ∼1800 nt insert was isolated from an adult stellate ganglion cDNA phage library. In addition, a library of about 23000 EST sequences that we obtained from the stellate ganglion of *Loligo pealei* (DeGiorgis et al., 2011) contained one entry with identical sequence to this cDNA, spanning nucleotides 240 to 652. Transcriptome sequences of the stellate ganglion and giant fiber system of this species fully matched the cloned sequence. One variant was always present in transcriptomes of *Loligo pealei*: a transcript that lacks a stretch of eight Ts in the untranslated 3′ end (underlined in Fig. 1). This cDNA had a predicted open reading frame from nucleotides 442 to 1443 (Fig. 1) as indicated by the translation initiation algorithm of NetStart 1.0 (http://www.cbs.dtu.dk/services/NetStart/). There was a variant Kozak sequence present at the translation initiation site 442 (CUCAUCAUGG) (Kozak, 1999). This protein had a length of 333 amino acids with the 60 amino acid homeodomain between residues 132 and 191 (Fig. 1).

**Fig. 2.**
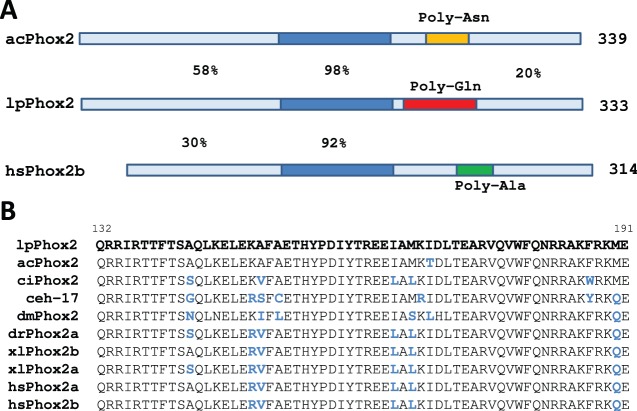
**Structural properties of lpPhox2 and phylogenetic comparison.** (A) Comparison of the architecture of lpPhox2B to human Phox2b (hsPhox2b) and Phox2 of *Aplysia californica* (acPhox2). Percentages of similarity are given for the homeodomain and the regions N-terminal and C-terminal of the homeodomain. Repeats of amino acids are indicated. (B) Comparison of the homeodomain of lpPhox2 with orthologs. The homeodomain sequences of the mollusk *Aplysia californica* (acPhox2), the ascidian *Ciona intestinalis* (ciPhox2), the nematode *C. elegans* (ceh-17), the fruit fly *Drosophila melanogaster* (dmPhox2), the zebra fish *danio rerio* (drPhox2a), and the two paralogs in the African claw toad *Xenopus leavis* (xlPhox2a/b) and man (*Homo sapiens*, hsPhox2a/b) are shown. Differences in amino acid residues are shown in blue.

Comparison of the 60-amino acid homeobox sequence to other invertebrates and vertebrates showed a high degree of conservation over a wide range of species, from 100% for *Sepia officinalis* to 92% for *Homo sapiens* (Fig. 2). The high degree of sequence similarity identified the predicted protein as the *Phox2* ortholog of *Loligo pealei*, *lpPhox2*. The cephalopod *Sepia officinalis* contained a predicted protein (accession: AGC24169) with 96% overall identity to lpPhox2. Differences were confined to a stretch of repeated Gln residues (Fig. 2). Orthologs of Phox2 were found in the cephalopodian mollusks *Euprymna scolopes* and *Lottia gigantea*. The mollusks *Aplysia californica* and *Lymnaea stagnalis* shared a 98% similarity in the homeodomain with lpPhox2. However, the overall homology was only 53% due to low similarity outside the homeodomain. Small patches of similarity were present in the N-terminal part of Phox2 proteins, specifically MEYxYLN (residues 1–7), GMDSS (residues 20–24) and TxGSCSxxxxxRDH (residues 62–75). Compared to vertebrate Phox2 proteins, lpPhox2 shared a strong homology in the homeobox (Fig. 2B) and the four amino acids preceding it (EKRK). Homology was also found in the extreme N-terminal motif MEYxYLN. The C-terminal domain lacked significant similarities (Fig. 2).

A lower degree of similarity in the homeodomain (88%) was found with Phox2 of other invertebrates, e.g. *C. elegans* (ceh-17) (Fig. 2). In the genome of the ctenophore *Mnemiopsis leidyi*, which is considered to belong to the most primitive metazoans (Marlow and Arendt, 2014), no evidence for a Phox2 ortholog was found. The most similar homeobox sequence to lpPhox2 rather appeared as an Aristaless ortholog. While vertebrates generally have two Phox2 paralogs, Phox2a and Phox2b, a single Phox2 sequence exists in databases of invertebrates. By mining *Loligo pealei* transcriptome and genome sequences, no evidence was obtained for Phox2 paralogs.

These data indicated that the squid *Loligo pealei* expresses a Phox2 transcription factor that is highly conserved in the homeodomain. The homeodomain confers the DNA-binding properties of this class of transcription factors, suggesting that the specificity of DNA-binding is a conserved, and, therefore, essential property for the function of Phox2 proteins.

### Transcriptional properties of lpPhox2

In view of the sequence divergence, we tested whether lpPhox2 could transcriptionally activate the human *dopamine beta-hydroxylase* (*DBH*) gene. The *DBH* promoter is an established mammalian Phox2 target gene which contains Phox2 response elements (Hwang et al., 2005; Kim et al., 1998). It has been shown that Phox2b and Phox2a of mouse, human and rat are transcriptional activators of the *DBH* gene. When a *DBH-luciferase* reporter construct was co-transfected with a mouse Phox2b expression plasmid in a heterologous cell system using Neuro2A cells, the *DBH* promoter activity was increased 10-fold. A *DBH* promoter-reporter construct with mutated response elements showed no significant change in promoter activity. However, when the mouse Phox2b expression plasmid was replaced by one for lpPhox2, *DBH* promoter activity was not influenced. These data show that lpPhox2 in contrast to mouse Phox2b lacks transcriptional activity in this heterologous mammalian cell system.

### Expression of lpPhox2

**Fig. 3.**
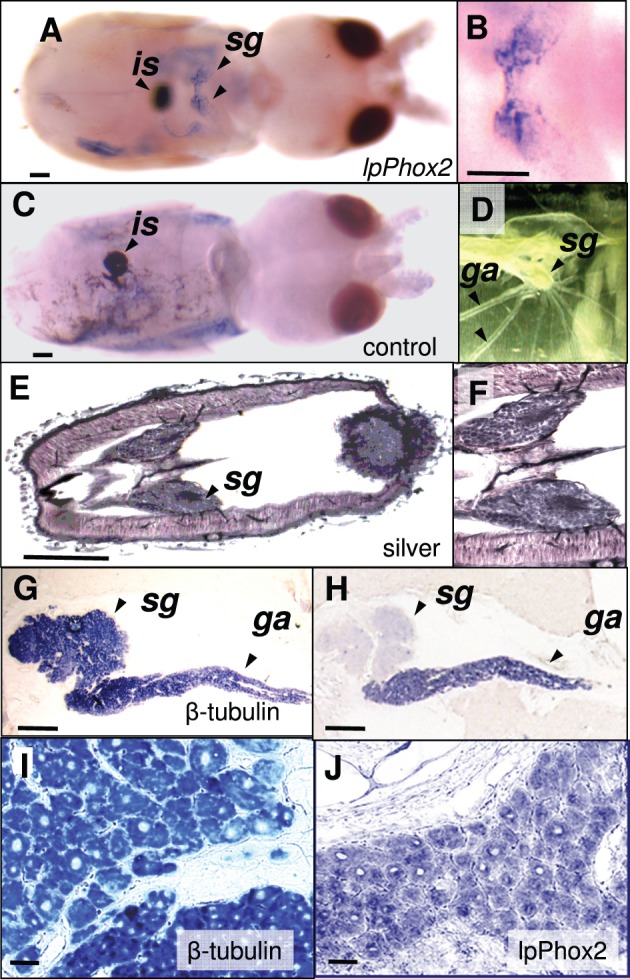
**Expression of lpPhox2 in the stellate ganglion of the squid *Loligo pealei*.** (A) Whole mount *in situ* hybridization of *lpPhox2* transcripts on a stage 27 embryo shows expression in the stellate ganglion (arrows). (B) Dorsal area of the embryo of panel A showing the pair of stellate ganglia. (C) Whole mount *in situ* hybridization on a stage 27 embryo using a lpPhox2 sense probe showing background staining. (D-F) Morphology of the stellate ganglion in an adult squid (D) and in a stage 27 embryo in a silver-stained paraffin section (E,F). (G) *In situ* hybridiation of the pan-neuronal gene *β-*tubulin shows expression in all parts of the adult stellate ganglion as demonstrated by *in situ* hybridization. (H) Selective expression of a gene coding for a hypothetical protein homologous to mouse KIAA1109 in the giant fiber lobe of the stellate ganglion. (I) Magnifications of panel G showing neuronal expression of *β-tubulin*. (J) Neuronal expression of *lpPhox2* in the adult giant fiber lobe. Abbreviations: *sg*, stellate ganglion; *ga*, giant axon; *is*, ink sac. Scale bars are 100 µm in panels A-E, 1 mm in G and H, and 10 µm in I and J.

The expression of *lpPhox2* was investigated in squid embryos and in the adult stellate ganglion by *in situ* hybridization and immunohistochemistry. Whole mount *in situ* hybridization on squid embryo of Arnold stage 27 (Arnold, 1990) revealed a signal in the stellate ganglion, while a sense probe did not show this staining (Fig. 3A,C). Whole mount *in situ* hybridization showed background staining in other head and body structures (Fig. 3). No *lpPhox2* mRNA signals were detected at earlier stages, although the stellate ganglion is present from stage 26 onwards. This suggested that *lpPhox2* was expressed during late embryonic development of the stellate ganglion. The stellate ganglion and giant fiber system is morphologically and functionally mature by stage 27, shown by giant axons leaving the ganglion to innervate mantle muscles (Fig. 3E,F) and the expression of phosphorylated neurofilament proteins (Grant et al., 1995). In fact, we observed that these hatchlings do exhibit a strong escape response.

In cryosections of stage 30 embryos weak signals of *lpPhox2* transcripts were observed in the central nervous system in scattered patterns. Staining was inconsistent due to the low signal (not shown). This indicated that lpPhox2 was expressed in brain structures, but at a low expression level. This observation agreed with a search in available transcriptomes of ganglia and brain lobe of *Loligo pealei* (http://ivory.idyll.org/blog/2014-loligo-transcriptome-data.html), showing the presence of lpPhox2 transcripts in the optical and buccal lobes, but not in the vertical lobe.

**Fig. 4.**
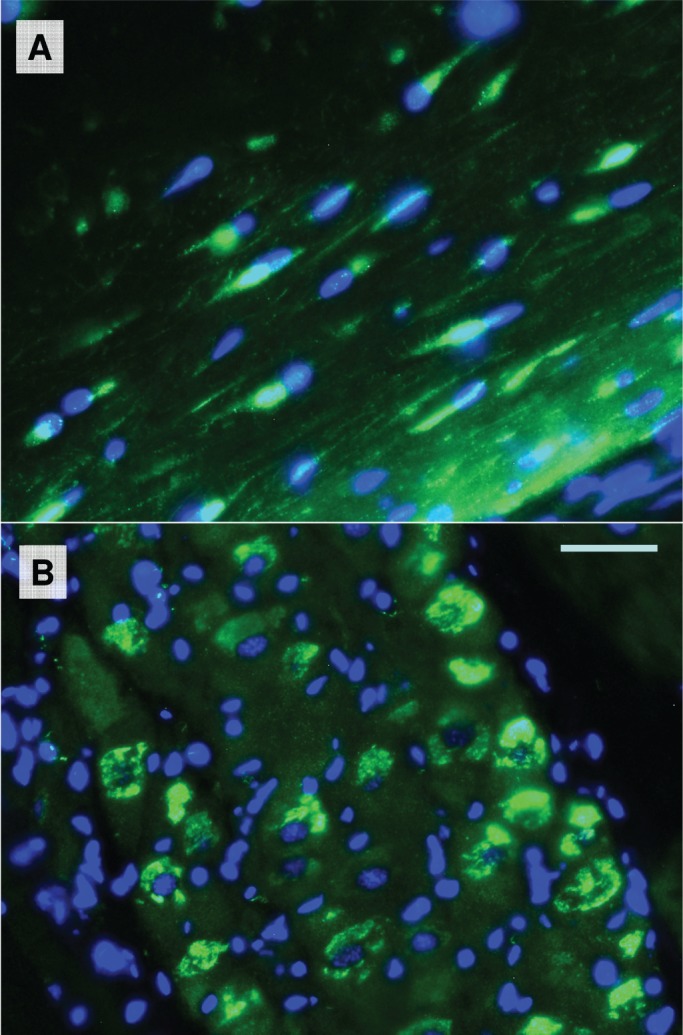
**Immunohistochemistry of lpPhox2 protein in the stellate ganglion.** Phox2 immunofluorescence is in green, nuclear staining by DAPI in blue. (A) All small neurons in the giant fiber lobe stain for Phox2-immunoreactivity. (B) Scattered large neurons in the central mass of the stellate ganglion stain immunoreactive for Phox2. The scale bar is 20 µm.

More intense signals of *lpPhox2* mRNA were obtained in the adult stellate ganglion (Fig. 3). We observed subregions of gene expression in the ganglion by *in situ* hybridization of β-tubulin (all neurons, and giant axon, Fig. 3G,I) and a transcript for a hypothetical protein homologous to mouse KIAA1109 (small giant fiber lobe neurons and giant axon, Fig. 3H). *lpPhox2* was expressed in small neurons in the giant fiber lobe (Figs 3J, 4A). Signals were far lower than those of β-tubulin (Fig 3H,J). Immunohistochemistry on isolated ganglia showed two types of lpPhox2 protein expression. One in nuclei of small neurons in the giant fiber lobe, and the other as non-nuclear staining in the central mass of the ganglion (Fig 4).

### Relationship to Fmrf expression

**Fig. 5.**
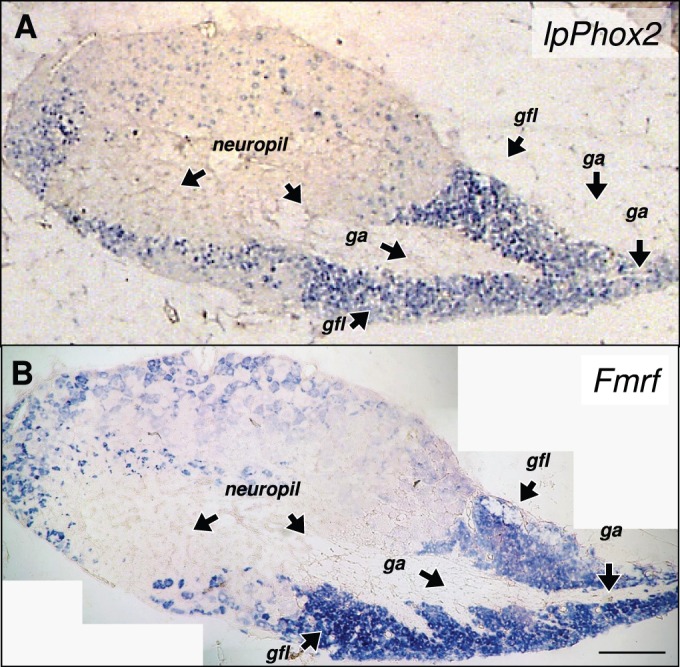
**Expression of the *lpPhox2* gene in the adult stellate ganglion and comparison to *Fmrf* gene expression.** (A) *In situ* hybridization of *lpPhox2* transcripts. Expression is highest in the small neurons of the giant fiber lobe. Individual axons from these neurons fuse to form the giant axons. (B) *In situ* hybridization of *Fmrf* transcripts, showing a similar distribution in the stellate ganglion. Panel A was a single micrograph, panel B was composed from multiple tiles. In A and B sections from the same ganglion were used. Abbreviations: *gfl*, giant fiber lobe; *ga*, giant axon. The scale bar is 0.5 mm.

We recently found that the *Fmrf* gene, coding for the neuropeptide FMRFamide, is highly expressed in the embryonic and adult stellate ganglion of *Loligo pealei* (Burbach et al., 2014). In the present study we noted similarities in the localization of lpPhox2 mRNA and *Fmrf* mRNA*.* Comparing the expression of *lpPhox2* and *Fmrf* in the adult ganglion by *in situ* hybridization showed that both genes displayed a similar distribution of expression in the adult stellate ganglion. Both genes were expressed predominantly by the small neurons of the giant fiber lobe (Fig 5).

**Fig. 6.**
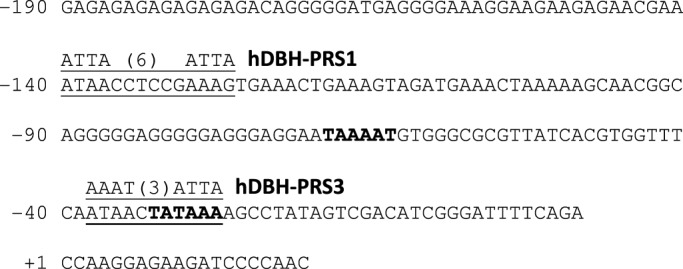
**Putative promoter region of the lpFMRF gene and regulatory elements.** The sequence was obtained from the *Loligo pealei* genome dump (http://ivory.idyll.org/blog/2014-loligo-transcriptome-data.html). In bold are two potential TATA boxes. Two elements with the characteristic signature (interspaced A/T quadruplets) of Phox2a/b binding sites in the human DBH promoter are indicated (Hwang et al., 2005).

To investigate a potential involvement of lpPhox2 in the regulation of expression of the *Fmrf* gene, we determined the 5′ end of Fmrf transcripts by searching transcriptome databases of *Loligo pealei* nervous system lobes and a genome data dump of this squid (http://athyra.idyll.org/~t/blast/ceph/). In this way a short putative promoter region could be identified that contained two potential TATA boxes closely together (Fig 6). This sequence overlapped with a motif that resembles a Phox2a binding site in the human *DBH* gene promoter (Hwang et al., 2005), suggesting that Phox2a may regulate the expression of the *Fmrf* gene directly. Unfortunately, this suggestion could not be further tested functionally since lpPhox2 is not transcriptionally active in heterologous mammalian expression systems.

## DISCUSSION

In many nervous systems homeodomain proteins play a developmental role in the differentiation and specification of neuronal systems. Moreover, they are engaged in regulatory processes when expression is sustained in the matured state. The latter function has not been explored extensively. In this study we identified seven paired-type homeodomain sequences expressed in the adult squid stellate ganglion. This suggests that regulatory roles may be executed by diverse homeodomain proteins of the paired-type family in developing and mature neurons of the stellate ganglion of the squid *Loligo pealei*.

A developmental role of paired-type homeobox genes homologous to the ones that we identified in the squid (e.g. *Pitx3*, *Msx*, and particularly *Phox2*), has been demonstrated in mammals. In mammalian systems two paralogs exist for *Phox2*: *Phox2a* and *Phox2b*. These exert complementary and concerted functions in a spatiotemporal relationship during the development of the neurons of the visceral nervous system (D'Autréaux et al., 2011). *Phox 2a* is coexpressed with *Phox2b* and specifies visceral nuclei in mid- and hindbrain in early development (Coppola et al., 2005). *Phox2b* operates as a master regulator in the determination of transmitter identity and connectivity, as in the specification of the noradrenergic phenotype (Brunet and Pattyn, 2002). In addition, *Phox2a* and *Phox2b* are required for development of cranial motor neurons. They guide the development of noradrenergic centers throughout development. They also participate in the patterned coding of the neuroepithelium, in proliferation, and in specification of noradrenalin synthesis, particularly by induction and regulation of the noradrenalin-synthesizing enzyme DBH. Their functions may expand into adult phases, as suggested by the control of *Phox2b* on *DBH* expression in rodents (Yang et al., 1998; Zhu et al., 2005b) and the truncation of axonal fibers in a *C. elegans* mutant for the *Phox2a/b* ortholog *ceh-17/cePhox2* (Pujol et al., 2000).

*lpPhox2* in squid may have such developmental functions. We observed that the expression of *Phox2* in the stellate ganglion is relatively late during development; only in stage 27 embryos we were able to detect lpPhox2 transcripts by whole mount *in situ* hybridization. In contrast, the phosphorylation of 220 HNF which marks the stellate fibers, is earlier. This begins at stage 25 and is mature by hatching (Grant et al., 1995). We cannot exclude, however, that *Phox2* is expressed outside the stellate ganglion in specific neuroepithelial structures, analogous to *Phox2a* expression in the mouse (Pattyn et al., 1997). Interestingly, it has been proposed that the function of *Phox2* is generically directed towards the specification of visceral neurons, both sensory and motoneurons, in all animal species, even in the ascidian *Ciona intestinalis* (D'Autréaux et al., 2011). These systems are particularly engaged in respiration. For *Sepia officinalis* it has been proposed that *Phox2* serves the generation of stellate ganglionic neurons for a respiratory function (Nomaksteinsky et al., 2013).

In this respect it is of relevance to note the apparent coexpression of *lpPhox2* and the *Fmrf* gene in small neurons of the giant fiber lobe of adult *Loligo pealei*. These neurons fuse their efferents into the giant axons that control mantle contraction, and enable physiological functions such as respiration and the escape response. The neurotransmitter of these neurons is not entirely certain; it has been hypothesized that glutamine and asparagine may be employed. We have shown that the *Fmr*f gene is expressed in these neurons, albeit the biologically active FMRF amide tetrapeptide is not produced due to differential precursor processing. Instead, the peptide is expressed in smaller fibers within the stellar nerves that innervate mantle muscles involved in respiration (Burbach et al., 2014). Several results from the present study suggest that the *Fmrf* gene is a target of *lpPhox2*. First, the onset of expression is timed similarly, around stage 27–28. Secondly, the location of expression overlaps for the two genes, and, thirdly, the putative promoter region of *lpFfmr* gene contains motifs that resemble Phox2a/b response elements of the *hDBH* promoter. However, this suggestion cannot be tested at present. Since lpPhox2 appeared to be functionally inactive in a mammalian cell lines, we need to develop a homologous expression system, which is not available at present.

A noted feature of all invertebrate Phox2 proteins is that sequence conservation is exclusive to the homeodomain. Such restricted similarities have also been observed for other homeodomain orthologs. For example, species comparison of the Pitx family of paired-type homeodomain proteins displays a similar homology restricted to the homeodomain. In *C. elegans*, it was shown that the phenotype caused by ablation of *Unc-30*, the *Pitx1/2/3* ortholog, was rescued by human *Pitx2*, while the homeodomain is the only conserved portion between UNc-30 and Pitx2 (Westmoreland et al., 2001). This indicates that the recognition between the homeodomain and the appropriate response elements is highly conserved. In addition to this strict conservation, non-conserved N- and C-terminal portions outside the homeodomain may still have 3-dimensional structural features appropriate to elicit generic transcriptional activity functions by interaction with other transcription components.

The results suggest that transcriptional codes for the developmental specification and regulation of neuronal systems are at least in part conserved between mammals and squid. lpPhox2 may serve analogous functions in the development of the stellate ganglion neurons of *Loligo pealei* as Phox2a/b does in the regulation of neuronal development in mammals. Such conservation may render this ganglion and the giant fiber system as a useful model for addressing specific questions concerning neuronal development.

## MATERIALS AND METHODS

### Animals

Specimens of the North Atlantic long-finned squid *Loligo pealei* were caught in Vineyard Sound and obtained through the Marine Resources Centre of the Marine Biological Laboratory, Woods Hole, MA, USA. Catch, care and use of animal complied with all institutional and national rules. Egg mass deposits were obtained from squid kept in tanks with running seawater at 20°C and staged according to Arnold (1990). Embryos were harvested in seawater, fixed in 70% ethanol and stored at −20°C.

### Degenerate RT-PCR amplification of homeodomain cDNAs

Total RNA was isolated from dissected squid stellate ganglia and subjected to RT-PCR using degenerate primers based on homologies within the homeodomain. Primer sequences, procedures and conditions were as described before (Asbreuk et al., 2002). PCR fragments were cloned and sequenced. PCR fragments encoding β-tubulin, a hypothetical protein homologous to mouse KIAA1109 and collagen were identified and cloned.

### cDNA libraries and cloning

Stellate ganglia were dissected from adult squids and stored at −80°C. Poly(A+) RNA was isolated, size selected and multiple libraries were made in the lambda-ZAP *Expres*s vector by unidirectional cloning of cDNAs using the kits of Stratagene. Phage libraries were screened by filter hybridization with ^32^P-labeled homeodomain fragments. Plasmids were excised from purified lambda clones and both strands were Sanger sequenced.

### *In situ* hybridization

Whole mount *in situ* hybridizations were performed according to Harland (1991) with modifications (Wacker et al., 2004) on embryos fixed in MEM-buffered paraformaldehyde and stored in methanol. Samples were rehydrated, treated with proteinase K and acetylated in triethanolamine-acetic anhydride before hybridization. After prehybridization for 3 h, samples were hybridized with 140 ng DIG-labeled RNA probe in 50% formamide, 5× SSC, 10 µg/ml torula RNA, 1.5% blocking buffer (Boehringer Mannheim), 5 mM EDTA, 0.1% Tween, pH 7.0, at 68°C overnight. Embryos were washed and treated with RNAse A as described. *In situ* hybridization with DIG-labelled RNA probes on cryosections was performed as described (Smidt et al., 2004). The Phox2 probe was a 449 nt fragment in the 5′ region of the *lpPhox2* cDNA. The Fmrf probe was a full-length *Fmrf* cDNA of *Loligo pealei* (Burbach et al., 2014). A probe of squid α-tubulin was obtained by PCR.

### Immunohistochemistry

Dissected adult stellate ganglia, embryos, and hatchlings were fixed in 4% paraformaldehyde and kept in phosphate-buffered saline with 30% sucrose for 2 days and cryosectioned at 16 μm. Immunocytochemistry was performed according to a previously published protocol (Grant et al., 1995). The primary antibody was a peptide-affinity purified rabbit polyclonal antibody raised against a synthetic peptide in the N-terminal part of human/mouse Phox2b [Phox2b antibody (center), AP11341c, Abgent]. This region is homologous to lpPhox2. The synthetic peptide was used for preabsorption of the antibody in controls for specificity. The secondary antibody was a donkey anti-rabbit Alexa-488 antibody (Life Technologies).

Photomicrographs were taken with a Zeiss Axio Imager Z2 system and images were adjusted for white balance, brightness, contrast and color level distribution using the Gimp open source software packages.

### Determination of promoter activity

Mouse Neuro2A cells, obtained from the American Type Culture Collection, were cultured in Dulbecco's modified Eagle's medium (GIBCO) supplemented with 4 mM L-glutamine, 20 U/ml penicillin, 20 μg/ml streptomycin, 1:100 (v/v) nonessential amino acids (GIBCO), and 10% fetal calf serum at 37°C in a 5% CO_2_/air mixture. Transfections were carried out in 6-well plates or 6-cm-diameter dishes by the calcium phosphate method; 1 μg of expression plasmid containing the coding region of mouse Phox2b or lpPhox2, plus 6.5 μg of luciferase reporter plasmid, and 16 μg/ml carrier plasmid [pSG5] were used for the 6-cm-diameter dishes and relative DNA amounts for the small-size 6-well plates. Reporter plasmids (kind gifts from K.S. Kim, Harvard University, Boston, MA, USA) contained the 5′ region of the human DBH gene (−978, −38, and four Phox2 response elements linked to the −38 region, fused to luciferase in pGL3 promoter (Kim et al., 1998). After 48 h the cells were harvested, and protein was extracted by lysis in 350 μl of lysis buffer: 1% Triton X-100, 0.1 M KH_2_PO_4_/K_2_HPO_4_ (pH 7.6), 15% glycerol, and 2 mM dithiothreitol. The lysate was used to assay luciferase activity.

## Supplementary Material

Supplementary Material

## References

[BIO012476C1] Andersson E., Jensen J. B., Parmar M., Guillemot F. and Björklund A. (2006). Development of the mesencephalic dopaminergic neuron system is compromised in the absence of neurogenin 2. *Development* 133, 507-516. 10.1242/dev.02224 16396906

[BIO012476C2] Arnold J. M. (1990). Embryonic development of the squid. In *Squid as Experimental Animals* (ed. Gilbert D. L., Adelman W. J. and Arnold J. M.), pp. 77-90. New York: Plenum Press.

[BIO012476C3] Asbreuk C. H. J., van Schaick H. S. A., Cox J. J., Smidt M. P. and Burbach J. P. H. (2002). Survey for paired-like homeodomain gene expression in the hypothalamus: restricted expression patterns of Rx, Alx4 and goosecoid. *Neuroscience* 114, 883-889. 10.1016/S0306-4522(02)00325-1 12379244

[BIO012476C4] Brunet J.-F. and Pattyn A. (2002). Phox2 genes — from patterning to connectivity. *Curr. Opin. Genet. Dev.* 12, 435-440. 10.1016/S0959-437X(02)00322-2 12100889

[BIO012476C5] Burbach J. P. H., Grant P., Hellemons A. J. C. G. M., Degiorgis J. A., Li K. W. and Pant H. C. (2014). Differential expression of the FMRF gene in adult and hatchling stellate ganglia of the squid Loligo pealei. *Biol. Open* 3, 50-58. 10.1242/bio.20136890 24326188 PMC3892160

[BIO012476C6] Coppola E., Pattyn A., Guthrie S. C., Goridis C. and Studer M. (2005). Reciprocal gene replacements reveal unique functions for Phox2 genes during neural differentiation. *EMBO J.* 24, 4392-4403. 10.1038/sj.emboj.7600897 16319924 PMC1356338

[BIO012476C7] Dasen J. S., Tice B. C., Brenner-Morton S. and Jessell T. M. (2005). A Hox regulatory network establishes motor neuron pool identity and target-muscle connectivity. *Cell* 123, 477-491. 10.1016/j.cell.2005.09.009 16269338

[BIO012476C8] D'Autréaux F., Coppola E., Hirsch M.-R., Birchmeier C. and Brunet J.-F. (2011). Homeoprotein Phox2b commands a somatic-to-visceral switch in cranial sensory pathways. *Proc. Natl. Acad. Sci. USA* 108, 20018-20023. 10.1073/pnas.1110416108 22128334 PMC3250195

[BIO012476C9] Davis S. W., Castinetti F., Carvalho L. R., Ellsworth B. S., Potok M. A., Lyons R. H., Brinkmeier M. L., Raetzman L. T., Carninci P., Mortensen A. H. et al. (2010). Molecular mechanisms of pituitary organogenesis: in search of novel regulatory genes. *Mol. Cell. Endocrinol.* 323, 4-19. 10.1016/j.mce.2009.12.012 20025935 PMC2909473

[BIO012476C42] DeGiorgis J. A., Cavaliere K. R. and Burbach J. P. H. (2011). Identification of molecular motors in the Woods Hole squid, Loligo pealei: an expressed sequence tag approach. *Cytoskeleton (Hoboken)* 68, 566-577. 10.1002/cm.20531 21913340

[BIO012476C10] Gilly W. F., Lucero M. T. and Horrigan F. T. (1990). Control of the spatial distribution of sodium channels in giant fiber lobe neurons of the squid. *Neuron* 5, 663-674. 10.1016/0896-6273(90)90220-A 2171590

[BIO012476C11] Goridis C. and Brunet J.-F. (1999). Transcriptional control of neurotransmitter phenotype. *Curr. Opin. Neurobiol.* 9, 47-53. 10.1016/S0959-4388(99)80006-3 10072363

[BIO012476C12] Grant P., Tseng D., Gould R. M., Gainer H. and Pant H. C. (1995). Expression of neurofilament proteins during development of the nervous system in the squid Loligo pealei. *J. Comp. Neurol.* 356, 311-326. 10.1002/cne.903560212 7629321

[BIO012476C13] Guillemot F. (2007). Cell fate specification in the mammalian telencephalon. *Prog. Neurobiol.* 83, 37-52. 10.1016/j.pneurobio.2007.02.009 17517461

[BIO012476C14] Hammock E. A. D., Eagleson K. L., Barlow S., Earls L. R., Miller D. M. and Levitt P. (2010). Homologs of genes expressed in Caenorhabditis elegans GABAergic neurons are also found in the developing mouse forebrain. *Neural Dev.* 5, 32 10.1186/1749-8104-5-32 21122108 PMC3006369

[BIO012476C15] Harland R. M. (1991). In situ hybridization: an improved whole-mount method for Xenopus embryos. *Methods Cell Biol.* 36, 685-695. 1811161 10.1016/s0091-679x(08)60307-6

[BIO012476C16] Hodgkin A. L. and Huxley A. F. (1952). Propagation of electrical signals along giant nerve fibres. *Proc. R. Soc. Lond. B. Biol. Sci.* 140, 177-183. 10.1098/rspb.1952.0054 13003922

[BIO012476C17] Hwang D.-Y., Hwang M. M., Kim H.-S. and Kim K.-S. (2005). Genetically engineered dopamine beta-hydroxylase gene promoters with better PHOX2-binding sites drive significantly enhanced transgene expression in a noradrenergic cell-specific manner. *Mol. Ther.* 11, 132-141. 10.1016/j.ymthe.2004.08.017 15585414

[BIO012476C18] Jessell T. M. (2000). Neuronal specification in the spinal cord: inductive signals and transcriptional codes. *Nat. Rev. Genet.* 1, 20-29. 10.1038/35049541 11262869

[BIO012476C19] Kim H. S., Seo H., Yang C., Brunet J. F. and Kim K. S. (1998). Noradrenergic-specific transcription of the dopamine beta-hydroxylase gene requires synergy of multiple cis-acting elements including at least two Phox2a-binding sites. *J. Neurosci.* 18, 8247-8260. 9763470 10.1523/JNEUROSCI.18-20-08247.1998 PMC6792837

[BIO012476C20] Kozak M. (1999). Initiation of translation in prokaryotes and eukaryotes. *Gene* 234, 187-208. 10.1016/S0378-1119(99)00210-3 10395892

[BIO012476C21] Lee P. N., Callaerts P., de Couet H. G. and Martindale M. Q. (2003). Cephalopod Hox genes and the origin of morphological novelties. *Nature* 424, 1061-1065. 10.1038/nature01872 12944969

[BIO012476C22] Llinas R. R. (1988). The intrinsic electrophysiological properties of mammalian neurons: insights into central nervous system function. *Science* 242, 1654-1664. 10.1126/science.3059497 3059497

[BIO012476C23] Marder E. (2002). Non-mammalian models for studying neural development and function. *Nature* 417, 318-321. 10.1038/417318a 12015611

[BIO012476C43] Marlow H. and Arendt D. (2014). Evolution: ctenophore genomes and the origin of neurons. *Curr. Biol.* 24, R757-R761. 10.1016/j.cub.2014.06.057 25137591

[BIO012476C24] Martin R. (1965). On the structure and embryonic development of the giant fibre system of the squid Loligo vulgaris. *Z. Zellforsch. Mikrosk. Anat.* 67, 77-85. 10.1007/BF00339277 5883168

[BIO012476C25] Molyneaux B. J., Arlotta P., Menezes J. R. L. and Macklis J. D. (2007). Neuronal subtype specification in the cerebral cortex. *Nat. Rev. Neurosci.* 8, 427-437. 10.1038/nrn2151 17514196

[BIO012476C26] Nomaksteinsky M., Kassabov S., Chettouh Z., Stoeklé H.-C., Bonnaud L., Fortin G., Kandel E. R. and Brunet J.-F. (2013). Ancient origin of somatic and visceral neurons. *BMC Biol.* 11, 53 10.1186/1741-7007-11-53 23631531 PMC3660236

[BIO012476C27] Pattyn A., Morin X., Cremer H., Goridis C. and Brunet J. F. (1997). Expression and interactions of the two closely related homeobox genes Phox2a and Phox2b during neurogenesis. *Development* 124, 4065-4075. 9374403 10.1242/dev.124.20.4065

[BIO012476C28] Pearson J. C., Lemons D. and McGinnis W. (2005). Modulating Hox gene functions during animal body patterning. *Nat. Rev. Genet.* 6, 893-904. 10.1038/nrg1726 16341070

[BIO012476C29] Pujol N., Torregrossa P., Ewbank J. J. and Brunet J. F. (2000). The homeodomain protein CePHOX2/CEH-17 controls antero-posterior axonal growth in C. elegans. *Development* 127, 3361-3371. 10887091 10.1242/dev.127.15.3361

[BIO012476C30] Sen S., Reichert H. and VijayRaghavan K. (2013). Conserved roles of ems/Emx and otd/Otx genes in olfactory and visual system development in Drosophila and mouse. *Open Biol.* 3, 120177 10.1098/rsob.120177 23635521 PMC3866872

[BIO012476C31] Smidt M. P. and Burbach J. P. H. (2007). How to make a mesodiencephalic dopaminergic neuron. *Nat. Rev. Neurosci.* 8, 21-32. 10.1038/nrn2039 17180160

[BIO012476C32] Smidt M. P., Smits S. M., Bouwmeester H., Hamers F. P. T., van der Linden A. J. A., Hellemons A. J. C. G. M., Graw J. and Burbach J. P. H. (2004). Early developmental failure of substantia nigra dopamine neurons in mice lacking the homeodomain gene Pitx3. *Development* 131, 1145-1155. 10.1242/dev.01022 14973278

[BIO012476C33] Trainor P. A. and Krumlauf R. (2001). Hox genes, neural crest cells and branchial arch patterning. *Curr. Opin. Cell Biol.* 13, 698-705. 10.1016/S0955-0674(00)00273-8 11698185

[BIO012476C34] Wacker S. A., McNulty C. L. and Durston A. J. (2004). The initiation of Hox gene expression in Xenopus laevis is controlled by Brachyury and BMP-4. *Dev. Biol.* 266, 123-137. 10.1016/j.ydbio.2003.10.011 14729483

[BIO012476C35] Webb D. A. and Young J. Z. (1940). Electrolyte content and action potential of the giant nerve fibres of loligo. *J. Physiol.* 98, 299-313. 10.1113/jphysiol.1940.sp003851 16995205 PMC1394034

[BIO012476C36] Westmoreland J. J., McEwen J., Moore B. A., Jin Y. and Condie B. G. (2001). Conserved function of Caenorhabditis elegans UNC-30 and mouse Pitx2 in controlling GABAergic neuron differentiation. *J. Neurosci.* 21, 6810-6819. 11517269 10.1523/JNEUROSCI.21-17-06810.2001 PMC6763078

[BIO012476C37] Yang C., Kim H.-S., Seo H., Kim C.-H., Brunet J.-F. and Kim K.-S. (1998). Paired-like homeodomain proteins, Phox2a and Phox2b, are responsible for noradrenergic cell-specific transcription of the dopamine beta-hydroxylase gene. *J. Neurochem.* 71, 1813-1826. 10.1046/j.1471-4159.1998.71051813.x 9798905

[BIO012476C38] Young J. Z. (1939). Fused neurons and synaptic contacts in the giant nerve fibres of cephalopods. *Philos. Trans. R. Soc. Lond. B Biol. Sci.* 229, 465-503. 10.1098/rstb.1939.0003

[BIO012476C39] Young J. Z. (1973). The giant fibre synapse of Loligo. *Brain Res.* 57, 457-460. 10.1016/0006-8993(73)90149-2 4722064

[BIO012476C40] Zhu X., Lin C. R., Prefontaine G. G., Tollkuhn J. and Rosenfeld M. G. (2005a). Genetic control of pituitary development and hypopituitarism. *Curr. Opin. Genet. Dev.* 15, 332-340. 10.1016/j.gde.2005.04.011 15917210

[BIO012476C41] Zhu M.-Y., Wang W.-P., Iyo A. H., Ordway G. A. and Kim K.-S. (2005b). Age-associated changes in mRNA levels of Phox2, norepinephrine transporter and dopamine beta-hydroxylase in the locus coeruleus and adrenal glands of rats. *J. Neurochem.* 94, 828-838. 10.1111/j.1471-4159.2005.03245.x 16033425 PMC2923405

