# Supplementary material for: The homeodomain transcription factor Phox2 in the stellate ganglion of the squid Loligo pealei
Source: Biol Open. 2015 Jun 26;4(8):954–60. doi: 10.1242/bio.012476 (PMC4542286; doi:10.1242/bio.012476)
Supplement: Supplementary Material [file supp_4_8_954__index.html]

The homeodomain transcription factor Phox2 in the stellate ganglion of the squid Loligo pealei — Supplementary Material 

# The homeodomain transcription factor Phox2 in the stellate ganglion of the squid *Loligo pealei*

## BIO012476 Supplementary Material

- Supplementary Material
