# Supplementary material for: The homeodomain transcription factor Phox2 in the stellate ganglion of the squid Loligo pealei
Source: Biol Open. 2015 Jun 26;4(8):954–60. doi: 10.1242/bio.012476 (PMC4542286; doi:10.1242/bio.012476)
Supplement: Supplementary Material [file supp_bio.012476_BIO012476supp.pdf]

The protein sequences were obtained by PCR amplification of cDNAs from Loligo pealei stellate ganglion RNA using degenerate primers, followed by comparison of obtained nucleotide sequences to the Loligo pealei transcriptome data base at http://athyra.idyll.org/~t/blast/ceph/

Phylogenetic relationships were determined using BLAST at http://blast.ncbi.nlm.nih.gov/Blast.cgi

lpNKX1-related protein 1

Predicted protein from *loligo pealei* transcriptome

```
NNNNNNNNSSNQQQAQQQRSQTTSFSVADILDPTKFTGTTSCIKTRVWHPWVDTSSTDSTDLSEKASDT
AESKDESIFEDACDKDDASGILEPDDKDVDESGKQKIKSFSSSGSDSGKAGKPRRARTAFTYEQLVAL
ENKFKTTRYLSVCERLNLALSLNLTETQVKIWFQNRRTKWKKQNPGMDVNSPTIPPSSGALSGFSSPY
SSTTPLYAQGLHPYLQNHNLYGALGLFRTHAASYGAQNHHIYYPYFSQTT
```

BLAST comparison of PCR amplified fragment

RecName: Full=NK1 transcription factor-related protein 1; AltName: Full=Homeobox protein 153; Short=HPX-153; AltName: Full=Homeobox protein SAX-2; AltName: Full=NKX-1.1
Sequence ID: sp|Q15270.2|NKX11_HUMANLength: 411Number of Matches: 1
Range 1: 250 to 324GenPeptGraphics Next Match Previous Match

Alignment statistics for match #1

Score	Expect	Method	Identities	Positives	Gaps
144 bits(364)	4e-39	Compositional matrix adjust.	68/76(89%)	72/76(94%)	1/76(1%)

```
Query  111  SGSDSGKAGKPRRARTAFTYEQLVALENKFKTTRYLSVCERLNLALSLNLTETQVKIWFQ  170
            +GSDS K+GKPRRARTAFTYEQLVALENKFK TRYLSVCERLNLALSL+LTETQVKIWFQ
Sbjct  250  TGSDS-KSGKPRRARTAFTYEQLVALENKFKATRYLSVCERLNLALSLSLTETQVKIWFQ  308

Query  171  NRRTKWKKQNPGMDVN  186
            NRRTKWKKQNPG D +
Sbjct  309  NRRTKWKKQNPGADTS  324
```

BLAST comparison of predicted protein to closest homolog

PREDICTED: GATA zinc finger domain-containing protein 7-like [Aplysia californica]
Sequence ID: ref|XP_005097511.1|Length: 771Number of Matches: 1
Range 1: 520 to 771GenPeptGraphics Next Match Previous Match

Alignment statistics for match #1

Score	Expect	Method	Identities	Positives	Gaps
256 bits(655)	6e-76	Compositional matrix adjust.	142/254(56%)	160/254(62%)	23/254(9%)

```
Query  22   TSFSVADILDPTKFTGTTSCIKTRVWHPWVDTSS----------TDSTDLSEKASDTAES  71
            T+FSVADILDP+KFTG     +  VW PW    S           D T     A+D  +
Sbjct  520  TAFSVADILDPSKFTGNGGGSRPPVWSPWQSQHSPGPGGPLAARADGTRSPGVAADARQG  579

Query  72   K----DESI------FEDACDKDDAS-GILEPDDKDVDESGKQKIKSFSSSGSDSGKAGK  120
            +    D SI      F+   D D    G     D+D DE  +    + + + SDS K GK
Sbjct  580  QRHDDDVSITSSQENFDPEEDVDMCEDGKHHTGDEDSDEEKRNADDTGNGNISDSSKQGK  639

Query  121  PRRARTAFTYEQLVALENKFKTTRYLSVCERLNLALSLNLTETQVKIWFQNRRTKWKKQN  180
            PRRARTAFTYEQLVALENKFKTTRYLSVCERLNLALSLNLTETQVKIWFQNRRTKWKKQN
Sbjct  640  PRRARTAFTYEQLVALENKFKTTRYLSVCERLNLALSLNLTETQVKIWFQNRRTKWKKQN  699

Query  181  PGMDVNSPTIPPSSGALSGFSSPYSSTTPLYAQGLHPYLQNHNLYGALGLFRTHAASYGA  240
            PG+DVNSPTIPP+SG    FSSPYSS   LY Q +HPYL    L   L + R H +
```

```
Sbjct  700   PGLDVNSPTIPPTSGGFGSFSSPYSSM--LYGQSIHPYLPASGLMSPLSILRAHGSYSPG  757

Query  241   QNHHIYYPYFSQTT  254
             QN  +YYPYFSQTT
Sbjct  758   QNPTVYYPYFSQTT  771
```

lpMSX2

Predicted protein from *loligo pealei* transcriptome

```
TAQKCTLRKHKTNRKPRTPFTTSQLLALERKFRTKQYLSIAERAEFSASLNLTETQVKIWFQNRRAKAKRLHEAE
LEKLKMAAKPMLPPALSITFPAAAALYGNQASRPQLIPHALLPPFGFYTTAPSTMIYHH
```

Msx protein, partial [Heterololigo bleekeri]
Sequence ID: dbj|BAF91577.1|Length: 114Number of Matches: 1
Range 1: 1 to 114GenPeptGraphics Next Match Previous Match
Alignment statistics for match #1

Score	Expect	Method	Identities	Positives	Gaps
228 bits(580)	3e-74	Compositional matrix adjust.	113/114(99%)	114/114(100%)	0/114(0%)

```
Query  21    TTSQLLALERKFRTKQYLSIAERAEFSASLNLTETQVKIWFQNRRAKAKRLHEAELEKLK  80
             TTSQLLALERKFRTKQYLSIAERAEFSASLNLTETQVKIWFQNRRAKAKRLHEAELEKLK
Sbjct  1     TTSQLLALERKFRTKQYLSIAERAEFSASLNLTETQVKIWFQNRRAKAKRLHEAELEKLK  60

Query  81    MAAKPMLPPALSITFPAAAALYGNQASRPQLIPHALLPPFGFYTTAPSTMIYHH  134
             MAAKPMLPPALSITFPAAAALYG+QASRPQLIPHALLPPFGFYTTAPSTMIYHH
Sbjct  61    MAAKPMLPPALSITFPAAAALYGSQASRPQLIPHALLPPFGFYTTAPSTMIYHH  114
```

MSX2 protein [Homo sapiens]
Sequence ID: dbj|BAA03611.1|Length: 172Number of Matches: 1
Range 1: 39 to 170GenPeptGraphics Next Match Previous Match
Alignment statistics for match #1

Score	Expect	Method	Identities	Positives	Gaps
185 bits(470)	1e-58	Compositional matrix adjust.	99/134(74%)	106/134(79%)	7/134(5%)

```
Query  5     CTLRKHKTNRKPRTPFTTSQLLALERKFRTKQYLSIAERAEFSASLNLTETQVKIWFQNR  64
             CTLRKHKTNRKPRTPFTTSQLLALERKFR KQYLSIAERAEFS+SLNLTETQVKIWFQNR
Sbjct  39    CTLRKHKTNRKPRTPFTTSQLLALERKFRQKQYLSIAERAEFSSSLNLTETQVKIWFQNR  98

Query  65    RAKAKRLHEAELEKLKMAAKPMLPPALSITFP-----AAAALYGNQASRPQLIPHALLPP  119
             RAKAKRL EAELEKLKMAAKPMLP + S+ FP      AA++YG  AS P    P   +PP
Sbjct  99    RAKAKRLQEAELEKLKMAAKPMLPSSFSLPFPISSPLQAASIYG--ASYPFHRPVLPIPP  156

Query  120   FGFYTTAPSTMIYH  133
              G Y T     +YH
Sbjct  157   VGLYATPVGYGMYH  170
```

lpZFHX4

Predicted protein from *loligo pealei* transcriptome

```
PPPPPPPMPPHHPPTSHGSSSQCAPVSIPSSQPSSSSSSSSSHMSEKVLSGQEMNLPQNPLDFTQMMPVFNMMHM
PVPMNMNLAMGMHPPLMPMMMPPSIESGFMPPMTMIDPNFLAAQQQAQHAQNQKRARTRINDEQLKILRANFDIN
NSPSEEQIVQMSEQSGLPQKVIKHWFRNTLFKERQRNKDSPYNFSNPPQTTINLEEYEKTGRIPSPSEQDLDNEF
KEELKSRYDDQLDDCDLQSRIDQVSSEADFPCTVTSVPMTFPPTLPIPSPLINHMPQTVPNHITSTPVGHRHIPM
EFQEKNDYFGNNSNNSNSSGSNNSQGGSGKRANRTRFTDYQIKVLQEYFEQNAYPKDDDLDHLSKLLNLSPRVIV
VWFQNARQKARKIYENQSPADLKDDGSGNFSRSGPNYQYQCKKCHSIFPRYYDLMKHQKRQNCQDGDKSGTSNMP
NDEDSNSTVTSHEETAHSESGEINAAELPKPISTIANSNCNSLKEVTATSTVMAIVPSGVATTTSAPVAISTTTS
SVLSTPPTPTPISCAGISNNLPTSLTSGSAGSSSSSSKTEIHKCEKCNLMFPKFEQWQEHQKVHSLNPNLFANFP
NDNTFGLLQNIAPTPPQQQPQLPPQHLQNMLHDKVMTPIKRKADEEEEERDQPRDKRLRTTILPEQLDYLYQKYQ
IDCNPSRKQLETIASEVGLKKRVVQVWFQNTRARERKGQYRAHQQLSHKRCPFCRALFRAKSALESHLATKHPEE
MAKGEINIDNIPDATVDSTPPPPPPPPPVSQAPSSSSLTGNPDLSKLLSPTNMQPFLPFMPQGNMGMGFTGLSDP
LQTSMRQFCEASFKKFINELSAASHGQRPQQPNVEPPQNKAAASEPEKKPARVIENKNSEDNDAPLDLSKPIRVN
TDCEKTSSDGPSTDVSERSMDGYLRRHSLDDSISETHSESHEIEGCEDSSGMIDNSPSSPANATSSSSSSSHPHG
KRYRTQMSSMQVRVMKTLFADYKTPTMAECEMLGREIGLPKRVVQVWFQNARAKEKKNKLAYSKTFGTDVDFSKP
PEECTLCNFKYSHKYTVQDHIFTKKHIDNVRQFIQSQSNAERELADPSGMTKMLQQQREIERMRKVWDEASVASS
PHLAQLQAMRLNALGLQGTSSAFNTPFNASSFFPDVKKEALKAEGEKKEKEETQSRRDAANFLSPQLMAPFTGLG
FEPSLLPNIYTGFPNFFSGVNLPLFQFGLMPGMEQLLAYDPVTFGTPLSLLQIPEQARRHVSAKLLEPSSVVAQY
TQDCKTLADLKSAVSDTDLKCVQESTVDVGYICKKCHIVYPGKDGCENHQHLICFPGGKVSDGIKPILKLEQVQY
ECVVCQEKFSTSQEFILHCSLELHKNKSTRHLSKSEWRLAPSLTPAPSENSAKEAKLPSVSMSTALTKKQSPGSD
KIPLPSSDSMASAAATTTPTPPTTTAAAAATALSSSVQTQNVLQLA
```

PREDICTED: zinc finger homeobox protein 3-like [Aplysia californica]
Sequence ID: ref|XP_005096810.1|Length: 2638Number of Matches: 8
Range 1: 1434 to 2515GenPeptGraphics Next Match Previous Match

Alignment statistics for match #1

Score	Expect	Method	Identities	Positives	Gaps
849 bits(2193)	0.0	Compositional matrix adjust.	531/1147(46%)	685/1147(59%)	163/1147(14%)

```
Query  329   GKRANRTRFTDYQIKVLQEYFEQNAYPKDDDLDHLSKLLNLSPRVIVVWFQNARQKARKI  388
             GKRANRTRFTD+QIK LQEYFE+NAYPKDD+L+HLSK+L LS RVIVVWFQNARQKARKI
Sbjct  1434  GKRANRTRFTDFQIKTLQEYFERNAYPKDDELEHLSKMLGLSARVIVVWFQNARQKARKI  1493

Query  389   YENQSPADLKDDGSGNFSRSGPNYQYQCKKCHSIFPRYYDLMKHQKRQNC--QDGDKSGT  446
             YENQ   ++  D +  F R+ P   YQCKKC+S+F RYY+L+KHQK  +C  +  +
Sbjct  1494  YENQPVNEIAKDPAVPFQRT-PGLNYQCKKCNSVFQRYYELIKHQK-NSCFGESNNNKPI  1551

Query  447   SNMPNDEDSNSTVTSHEETAHSESGEINAAELPKPISTIANSNCNSLKEVTATSTVMAIV  506
             S+M  D+ SN +  S+E++  S+                        + + +++T
Sbjct  1552  SSMTEDDSSNISYNSNEDSNFSD------------------------RHIPSSATP----  1583

Query  507   PSGVATTTSAPVAISTTTSSVLSTPPTPTPISCAGISNNLPTSLTSGSAGSSSSSSKTEI  566
                V      P A++  T+S+ +TP  P  +S + ++    TS +S S  SSSSSS
Sbjct  1584  ---VRERELMPRALAVETTSISTTPAKPV-LSASAVN----TSSSSPSTPSSSSSSSNPP  1635

Query  567   HKCEKCNLMFPKFEQWQEHQKVHSLNPNLFANFPNDNTFGLLQNIA--------------  612
              KC+KC L FP+F+ WQ+HQK HS+ P +F  F + + FG+LQ +A
Sbjct  1636  FKCDKCTLTFPRFDMWQDHQKAHSVAPAMFTPFSSSSAFGMLQTLAHQEDSKAVFAMAAM  1695

Query  613   PTPPQQQPQL-----PPQH---LQNMLHDKVMTP------IKRKADEEEEERDQPRDKRL  658
              TP    P +     P  H       L   VM+        KRK D E+E  +QPRDKRL
Sbjct  1696  STPSSMHPTMVTTPIPAAHQPPAMTTLPTSVMSTSPSPATTKRKTDSEDESGEQPRDKRL  1755

Query  659   RTTILPEQLDYLYQKYQIDCNPSRKQLETIASEVGLKKRVVQVWFQNTRARERKGQYRAH  718
             RTTILPEQLDYLYQ+YQIDCNPSRKQLE IAS V LKKRVVQVWFQNTRARERKG YRAH
Sbjct  1756  RTTILPEQLDYLYQQYQIDCNPSRKQLEHIASTVNLKKRVVQVWFQNTRARERKGHYRAH  1815

Query  719   QQLSHKRCPFCRALFRAKSALESHLATKHPEEMAKGEINIDNIPDATVDSTPPPPPPPPP  778
```

```
             QQL +KRCPFCRALFRAKSALESHLATKHPEEMAKG+IN+D IPDA ++        PP
Sbjct  1816  QQLINKRCPFCRALFRAKSALESHLATKHPEEMAKGDINVDLIPDAMIE--------PPS  1867

Query  779   VSQAPS----SSSLTGN---PDLSKLLSP---TNMQPFLPFM--PQGNMGMGFTGLSDPL  826
              S APS    SS LT     PD+SKLL P    +M  ++ FM    G + + F   +  L
Sbjct  1868  SSHAPSLPASSSGLTSTHNTPDISKLLPPGAAASMPNYMSFMSAAAGGLSLPFPAPTADL  1927

Query  827   QTSMRQFCEASFKKFINELSAASHGQRPQQPNVEPPQ-----------NKAAASEPEK--  873
               S   F +  FKK+++EL A +   RP+  +V PP            + +A S P+
Sbjct  1928  -LSHPSFEDPFFKKYMSEL-AGTMTSRPE--SVAPPLPHSMPRHSASPSISAHSTPKPSH  1983

Query  874   --------------KPARVIENKNS------EDNDAPLDLSKPIRVNTDCEKTSSDGPST  913
                           +P    + K S         DAPLDLSKP+R        +   P +
Sbjct  1984  HSSPAPLNFATTSPRPLDAHKVKTSTPIPPPASEDAPLDLSKPVRSAHPEPSRTEQVPRS  2043

Query  914   ---------DVSERSMD-GYLRR-HSLDDSISETHSESHEIEGCEDSSGMIDNSPSSPAN  962
                      + S+RS++  YLRR +S+DDS SET SE+ + E   +   + ++ PS   +
Sbjct  2044  FALHHSSGQEQSDRSLELDYLRRLNSMDDSFSETQSETADHEYLNE---VNNSPPSPNRS  2100

Query  963   ATSSSSSSSHPHG-------KRYRTQMSSMQVRVMKTLFADYKTPTMAECEMLGREIGLP  1015
             ++SS+   +H  G       KRYRTQMS+ QV+VMK LF DYKTPTMAECE+LG+EIGL
Sbjct  2101  SSSSNPPQNHRSGNTPGSSTKRYRTQMSATQVKVMKHLFMDYKTPTMAECELLGQEIGLA  2160

Query  1016  KRVVQVWFQNARAKEKKKNKLAYSKTFGTDVDFSKPPEECTLCNFKYSHKYTVQDHIFTKK  1075
             KRVVQVWFQNARAKEKK KL+  K F  ++DF K PEEC+LC +KY+HK TVQDHIFTK
Sbjct  2161  KRVVQVWFQNARAKEKKAKLSSGKPFNAELDFPKSPEECSLCGYKYTHKVTVQDHIFTKA  2220

Query  1076  HIDNVRQFIQSQSNAERELADPSGMTKMLQQQREIERMRKVWDEASVASSPHLAQLQAMR  1135
             HID V+ F+   ++ ER+L D SG +   Q   E ER RK+WDE    S  HLAQLQAM
Sbjct  2221  HIDKVKNFL--SADGERDL-DLSGGSLPSQSSAETERARKMWDE----SGTHLAQLQAMG  2273

Query  1136  L--NALGLQGTSSAFNTPFNASSFFPDVKKEALKAEGEKKEKEETQSRRDAANFLSPQLM  1193
             +  +A+G     S  ++  +  S  P  KK+   AE     KE+ Q   + A  +S Q+M
Sbjct  2274  ISPSAIGFPPLPSGESSKAHEKSESP--KKDKKPAEA-GSSKEQQQVAMEMA--MSAQMM  2328

Query  1194  APFTGL--GFEPSLLPNIYTGFPNFFSGVNLPLFQFGLMPGMEQLLAYDPVTFGTPLSLL  1251
             +   G   G +PS L  +Y G P +F  + +P+ Q GL+ G E ++ YDP+ +GTPL+LL
Sbjct  2329  SALGGYMPGLDPSYLSYMYGGLPGYFPAMGVPMVQPGLLSGAEHMMGYDPLAYGTPLALL  2388

Query  1252  QIPEQARRHVSAKLLEPSSVVAQYTQDCKTLADLKSAVSDTDLKCVQESTVDVGYICKKC  1311
             QIP  A + VS KL E  +V+A+YTQDC+ LADL S V+  DL    E+T+DVGYICK C
Sbjct  2389  QIPSAAIKSVSDKLSEAGAVLARYTQDCQALADLHSVVTAADLTVAAEATLDVGYICKTC  2448

Query  1312  HIVYPGKDGCENHQHLICFPG-GKVSDGIKPILKLEQVQYECVVCQEKFSTSQEFILHCS  1370
              IVYP ++ C  HQ  +C  G      G +PI+KLEQVQYEC  C ++FST  EF  HC
Sbjct  2449  QIVYPARESCIAHQRTVCGTGQTNRPKGFEPIMKLEQVQYECRACNDRFSTILEFKGHCQ  2508

Query  1371  LELHKNK  1377
              E HK +
Sbjct  2509  QEAHKTR  2515
```

Zinc finger homeobox protein 4 [Homo sapiens]

Sequence ID: dbj|BAD18546.1|Length: 907Number of Matches: 5

Range 1: 2 to 392GenPeptGraphics Next Match Previous Match

Alignment statistics for match #1

Score	Expect	Method	Identities	Positives	Gaps
204 bits(518)	1e-52	Compositional matrix adjust.	154/406(38%)	197/406(48%)	83/406(20%)

```
Query  413   QYQCKKCHSIFPRYYDLMKHQKRQNC---QDGDKSGTSNMPNDEDSNSTVTSHEETAHSE  469
             QYQCKKC+ +FPR +DL+ HQK+Q C   +D D    S   +  D+   V     T   +
Sbjct  2     QYQCKKCNVVFPRIFDLITHQKKQ-CYKDEDDDAQDESQTEDSMDATDQVVYKHCTVSGQ  60

Query  470   -------------SGEINAAEL---PKP----ISTIANSNCNSLKEVTATSTVMAIVPS-  508
```

```
                         SG   +  L   PKP     S          K+   +       P+
Sbjct  61   TDAAKNAAAPAASSGSGTSTPLIPSPKPEPEKTSPKPEYPAEKPKQSDPSPPSQGTKPAL  120

Query  509  GVATTTSAPVAISTT----------TSSVLSTPP----TPTP-----ISCAGISNNLPTS  549
             +A+T+S P   ST              ++  PP    TP P     IS   + N+LP
Sbjct  121  PLASTSSDPPQASTAQPQPQPQPPKQPQLIGRPPSASQTPVPSSPLQISMTSLQNSLPPQ  180

Query  550  LTSGSAGSSSSSSKTEIHKCEKCNLMFPKFEQWQEHQKVHSL---NPNLFANF---PNDN  603
            L                ++C++C + FP  E WQEHQ VH L   N  L + F   P D
Sbjct  181  LLQ--------------YQCDQCTVAFPTLELWQEHQHVHFLAAQNQFLHSPFLERPMDM  226

Query  604  TFGLLQNIAPTPPQQ-----QPQLPPQ--HLQNMLHDKVMTPIKRKADEEEEE-------  649
             + +     P    Q       Q+PPQ           V   +KRK D++E+
Sbjct  227  PYMIFDPNNPLMTGQLLGSSLTQMPPQASSSHTTAPTTVAASLKRKLDDKEDNNCSEKEG  286

Query  650  ----RDQPRDKRLRTTILPEQLDYLYQKYQIDCNPSRKQLETIASEVGLKKRVVQVWFQN  705
                 DQ RDKRLRTTI PEQL+ LY+KY +D NP+RK L+ IA EVGLKKRVVQVWFQN
Sbjct  287  GNSGEDQHRDKRLRTTITPEQLEILYEKYLLDSNPTRKMLDHIAREVGLKKRVVQVWFQN  346

Query  706  TRARERKGQYRA-HQQLSHKRCPFCRALFRAKSALESHLATKHPEE  750
            TRARERKGQ+RA     SHKRCPFCRALF+AKSALESH+ ++H  E
Sbjct  347  TRARERKGQFRAVGPAQSHKRCPFCRALFKAKSALESHIRSRHWNE  392
```

lpGSC/lpArx

```
MKGGREGWLVASKRGKERQNEQIRMMEKQQDCSVSGNVSITENARGSIVCSSSKRPPPCAGTLAESPH
LHSMQGSYSINGLLASHEGQTLGDNPSPRGTDTGLTNGFNSAKYLGYSSQSPVATAAAAAALSSKENQ
TSSTGNGEYQREPSSVGLNHYSDIPYGQQQVKRKQRRYRTTFTSYQLEELEKAFHKTHYPDVFCREEL
ALRIDLTEARVQVWFQNRRAKWRKQQKQQGKDASITQVLHSANAKCGVGSGVGGQGPSGVGTTLSGLS
LPNIPPLPNMALPSMYFHSNMAAAAGMDWPGPPIGGSLAPPQTSMASYMTTPGASAKCDNRLLHSSSE
MPTSIHGLKPVGLLNNQ
```

Aristaless-like homeobox protein, partial [Sepia officinalis]
Sequence ID: gb|AHY00644.1|Length: 217Number of Matches: 1

Alignment statistics for match #1

Score	Expect	Method	Identities	Positives	Gaps
381 bits(978)	4e-129	Compositional matrix adjust.	196/216(91%)	199/216(92%)	1/216(0%)

```
Query  2    KGGREGWLVASKRGKERQNEQIRMMEKQQDCSVSGNVSITENARGSIVCSSSKRPPPCAG  61
            +GG    LV S    ER NEQI  MEKQ DCS+SGNVSI E+ARGSI CSSSKRPPPC G
Sbjct  3    EGGEGRCLVVSNGRNERLNEQIGKMEKQ-DCSISGNVSIAEDARGSIACSSSKRPPPCTG  61

Query  62   TLAESPHLHSMQGSYSINGLLASHEGQTLGDNPSPRGTDTGLTNGFNSAKYLGYSSQSPV  121
            TLAESPHLHSMQGSYSINGLLASHEGQTLGDNPSPRGTDTGLTNGFNSAKYLGYSSQSPV
Sbjct  62   TLAESPHLHSMQGSYSINGLLASHEGQTLGDNPSPRGTDTGLTNGFNSAKYLGYSSQSPV  121

Query  122  ATAAAAAALSSKENQTSSTGNGEYQREPSSVGLNHYSDIPYGQQQVKRKQRRYRTTFTSY  181
            ATAAAAAALSSKENQTSSTGNGEYQRE SSVGLNHYSDIPYGQQQVKRKQRRYRTTFTSY
Sbjct  122  ATAAAAAALSSKENQTSSTGNGEYQREQSSVGLNHYSDIPYGQQQVKRKQRRYRTTFTSY  181

Query  182  QLEELEKAFHKTHYPDVFCREELALRIDLTEARVQV  217
            QLEELEKAFHKTHYPDVFCREELALRIDLTEARVQV
Sbjct  182  QLEELEKAFHKTHYPDVFCREELALRIDLTEARVQV  217
```

homeobox protein ARX [Homo sapiens]
Sequence ID: ref|NP_620689.1|Length: 562Number of Matches: 1

Alignment statistics for match #1

Score	Expect	Method	Identities	Positives	Gaps
135 bits(341)	3e-34	Compositional matrix adjust.	68/102(67%)	77/102(75%)	2/102(1%)

```
Query  130  LSSKENQTSSTGNGEYQREPSSVGLNHYSDIPYGQQQVKRKQRRYRTTFTSYQLEELEKA  189
            LS KE       + E +    SV L+  SD   G   +KRKQRRYRTTFTSYQLEELE+A
Sbjct  289  LSPKEELLLHPEDAEGKDGEDSVCLSAGSDSEEGL--LKRKQRRYRTTFTSYQLEELERA  346

Query  190  FHKTHYPDVFCREELALRIDLTEARVQVWFQNRRAKWRKQQK  231
            F KTHYPDVF REELA+R+DLTEARVQVWFQNRRAKWRK++K
Sbjct  347  FQKTHYPDVFTREELAMRLDLTEARVQVWFQNRRAKWRKREK  388
```

lpPOU6-2

Predicted protein from *loligo pealei* transcriptome

```
VLPPTTVSQQPTATRITVAAQSITSHTTTSEGLPPNVSQLLPNTVSNSTESTTVDGINLDEIKEFAKQFKIRRLS
LGLTQTQVGQALSATEGPAYSQSAICRFEKLDITPKSAQKIKPVLERWMAEAEERYKNGVQNLTDFIGSEPSKKR
KRRTSFTPQALEILNQHFEKNTHPSGAELTELSENLSYDREVVRVWFCNKRQALKNTIKLIRIF
```

PREDICTED: POU domain, class 6, transcription factor 1-like [Aplysia californica]
Sequence ID: ref|XP_005107010.1|Length: 432Number of Matches: 1
Range 1: 240 to 428GenPeptGraphics Next Match Previous Match

Alignment statistics for match #1

Score	Expect	Method	Identities	Positives	Gaps
360 bits(923)	4e-120	Compositional matrix adjust.	169/189(89%)	182/189(96%)	1/189(0%)

```
Query  25   SHTTTS-EGLPPNVSQLLPNTVSNSTESTTVDGINLDEIKEFAKQFKIRRLSLGLTQTQV  83
            SH ++S  GLPPNVSQLLP+TV NSTESTTVDGINLDEIKEFA+QFKIRRLSLGLTQTQV
Sbjct  240  SHISSSTNGLPPNVSQLLPHTVGNSTESTTVDGINLDEIKEFARQFKIRRLSLGLTQTQV  299

Query  84   GQALSATEGPAYSQSAICRFEKLDITPKSAQKIKPVLERWMAEAEERYKNGVQNLTDFIG  143
            GQALSATEGPAYSQSAICRFEKLDITPKSAQKIKPVLERWMAEAEERYKNGVQNLTDFIG
Sbjct  300  GQALSATEGPAYSQSAICRFEKLDITPKSAQKIKPVLERWMAEAEERYKNGVQNLTDFIG  359

Query  144  SEPSKKRKRRTSFTPQALEILNQHFEKNTHPSGAELTELSENLSYDREVVRVWFCNKRQA  203
            SEPSKKRKRRTSFTPQALE+LNQ+FE+NTHP+G E+TELSE L+YDREV+RVWFCNKRQA
Sbjct  360  SEPSKKRKRRTSFTPQALELLNQYFERNTHPTGTEMTELSERLNYDREVIRVWFCNKRQA  419

Query  204  LKNTIKLIR  212
            LKNTIK ++
Sbjct  420  LKNTIKKLK  428
```

POU domain, class 6, transcription factor 2 [Homo sapiens]
Sequence ID: gb|EAL23992.1|Length: 647Number of Matches: 1
Range 1: 453 to 626GenPeptGraphics Next Match Previous Match

Alignment statistics for match #1

Score	Expect	Method	Identities	Positives	Gaps
307 bits(787)	2e-99	Compositional matrix adjust.	144/177(81%)	161/177(90%)	3/177(1%)

```
Query  36   NVSQLLPNTVSNSTESTTVDGINLDEIKEFAKQFKIRRLSLGLTQTQVGQALSATEGPAY  95
            +V QL+ N   + + E    VDG+NL+EI+EFAK FKIRRLSLGLTQTQVGQALSATEGPAY
Sbjct  453  SVGQLVSNPQTAAGE---VDGVNLEEIREFAKAFKIRRLSLGLTQTQVGQALSATEGPAY  509

Query  96   SQSAICRFEKLDITPKSAQKIKPVLERWMAEAEERYKNGVQNLTDFIGSEPSKKRKRRTS  155
            SQSAICRFEKLDITPKSAQKIKPVLERWMAEAE R++ G+QNLT+FIGSEPSKKRKRRTS
Sbjct  510  SQSAICRFEKLDITPKSAQKIKPVLERWMAEAEARHRAGMQNLTEFIGSEPSKKRKRRTS  569

Query  156  FTPQALEILNQHFEKNTHPSGAELTELSENLSYDREVVRVWFCNKRQALKNTIKLIR  212
            FTPQALEILN HFEKNTHPSG E+TE++E L+YDREVVRVWFCNKRQALKNTIK ++
Sbjct  570  FTPQALEILNAHFEKNTHPSGQEMTEIAEKLNYDREVVRVWFCNKRQALKNTIKRLK  626
```
